# (*R*)-4-Isopropyl-3-isopropyl­sulfanyl-5,5-diphenyl-1,3-oxazolidin-2-one

**DOI:** 10.1107/S160053681202569X

**Published:** 2012-06-13

**Authors:** Gustavo Pozza Silveira, Cassandra Bonfante de Carvallho, Allen Oliver

**Affiliations:** a Departamento de Química, Instituto de Química, Universidade Federal do Rio, Grande do Sul, Porto Alegre/RS 91501-970, Brazil; b Department of Chemistry and Biochemistry, University of Notre Dame, Notre, Dame, IN 46556-5670, USA

## Abstract

The title compound, C_21_H_25_NO_2_S, consists of a five-membered heterocyclic ring, with pendant phenyl groups, an isopropyl group and a thio­ether residue. The thio­ether bonds to the heterocycle *via* the N atom. The absolute configuration results from an inversion of the configuration of substrate during the synthesis.

## Related literature

For background to the preparation of chiral auxiliaries containing sulfilimine functionalities, see: Celentano *et al.* (1998 ▶). For a related structure, see: Valle *et al.* (1992 ▶). For the synthesis, see: Hinter­mann & Seebach (1998 ▶); Derbesy & Harpp (1995 ▶). For the structural characterization and absolute configuration analysis, see: Flack (1983 ▶); Hooft *et al.* (2008 ▶). For a description of the Cambridge Structural Database, see Allen (2002 ▶).
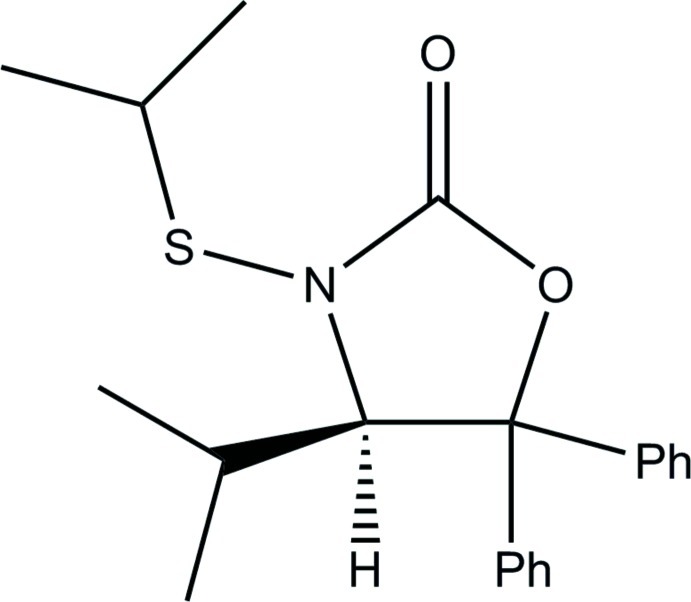


## Experimental

### 

#### Crystal data

C_21_H_25_NO_2_S
*M* *_r_* = 355.48
Orthorhombic, 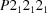

*a* = 6.0621 (1) Å
*b* = 17.2963 (3) Å
*c* = 18.5398 (3) Å
*V* = 1943.93 (6) Å^3^
*Z* = 4
Cu *K*α radiation
μ = 1.58 mm^−1^
*T* = 100 K
0.50 × 0.23 × 0.21 mm

#### Data collection

Bruker SMART APEX diffractometer
Absorption correction: numerical (*SADABS*; Sheldrick, 2008 ▶) *T*_min_ = 0.720, *T*_max_ = 0.964
18292 measured reflections
3008 independent reflections
2887 reflections with *I* > 2σ(*I*)
*R*_int_ = 0.026

#### Refinement

*R*[*F* ^2^ > 2σ(*F* ^2^)] = 0.027
*wR*(*F* ^2^) = 0.071
*S* = 1.06
3008 reflections
230 parameters
H-atom parameters constrained
Δρ_max_ = 0.19 e Å^−3^
Δρ_min_ = −0.16 e Å^−3^
Absolute structure: Flack (1983 ▶), 1165 Friedel pairs
Flack parameter: 0.039 (15)

### 

Data collection: *APEX2* (Bruker, 2008 ▶); cell refinement: *SAINT* (Bruker, 2008 ▶); data reduction: *SAINT*; program(s) used to solve structure: *SHELXS97* (Sheldrick, 2008 ▶); program(s) used to refine structure: *SHELXL97* (Sheldrick, 2008 ▶); molecular graphics: *XP* in *SHELXTL* (Sheldrick, 2008 ▶); software used to prepare material for publication: *publCIF* (Westrip, 2010 ▶).

## Supplementary Material

Crystal structure: contains datablock(s) I, global. DOI: 10.1107/S160053681202569X/tk5109sup1.cif

Structure factors: contains datablock(s) I. DOI: 10.1107/S160053681202569X/tk5109Isup2.hkl

Supplementary material file. DOI: 10.1107/S160053681202569X/tk5109Isup3.cml

Additional supplementary materials:  crystallographic information; 3D view; checkCIF report

## Figures and Tables

**Fig. 1.**
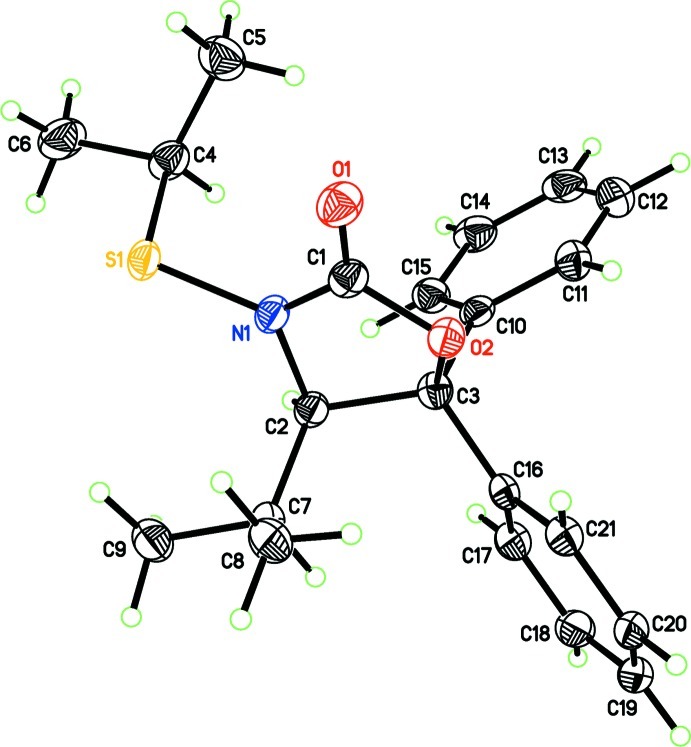
The molecular structure of (I) showing the atom-labelling scheme and displacement ellipsoids at the 50% probability level. H atoms are shown as idealized spheres of an arbitrary radius.

## supplementary crystallographic information

### Comment

Oxazolindinone compounds, such as the title compound, (*R*)-4-isopropyl-3-(isopropylthio)-5,5-diphenyloxazolidin-2-one (I), are synthesized as precursors for the preparation of chiral auxiliaries containing sulfilimine functionalities. Eventually, these auxiliaries are applied to the synthesis of new sulfimines in a high enantiomeric ratio (Celentano *et al.*, 1998). To the best of our knowledge, the only other *N*-thioether-containing oxazolindinone is a dione (Valle *et al.*, 1992). All other oxazoldininones that exhibit an N—S bond are sulfinyl- or sulfonyl-containing compounds (Allen, 2002).

An interesting feature of this compound is the conversion of *S*-isopropyl isopropanesulfonothioate to an *R*-isomer during the synthesis. Confirmation of the correct absolute stereochemistry of (I) was determined as described below.

### Experimental

To a solution of the oxazolidinone (Hintermann & Seebach, 1998) (2.50 g, 8.80 mmol) in dry THF (40 ml) at 273 K was slowly added 1 equiv of *n*-BuLi (Celentano *et al.*, 1998). The solution turned from colorless to dark-red. After the mixture was left to react for 30 min at 273 K, a solution of *S*-isopropyl isopropanesulfonothioate (Derbesy & Harpp, 1995) (1.58 g, 9.10 mmol) in dry THF (40 ml) was added by cannula, at once, and the reaction was left stirring overnight at room temperature. The white mixture was quenched with saturated NH_4_Cl (50 ml) and extracted with ethyl acetate (50 ml). The organic layer was washed with H_2_O (50 ml) and brine, dried with MgSO_4_ and then filtered. The solvent was removed at reduced pressure on a rotovap and the colorless oil was purified through flash chromatography with elution by (1:9 ethyl acetate/hexanes) to provide 2.28 g of the oxazolidine sulfide (73% yield) as colorless prisms after slow evaporation.

### Refinement

All hydrogen atoms were included in geometrically calculated positions with C—H distances constrained to 0.95 Å for aromatic C–H and 0.98–1.00 Å for aliphatic C–H bonds. Hydrogen thermal parameters were tied to the occupancy of the atom to which they are bonded. The *U*_iso_ was set to 1.5 × *U*_eq_ for methyl H atoms and 1.2 × *U*_eq_ for all others.

The absolute configuration was determined by the known handedness of the molecule from synthesis, comparison of intensities of Friedel pairs of reflections (Flack, 1983) and by Bayesian analysis of Bijvoet pairs (Hooft *et al.*, 2008). All three techniques agree and the correct configuration is depicted in Fig. 1. The Flack *x* parameter refined to 0.039 (15) based on 1165 Friedel pairs. The Hooft *y* parameter was 0.056 (6) based on 1170 Bijvoet pairs. P2(true) and P3(true) values were calculated at 1.000 and 1.000 indicative an an enantiopure crystal.

### Crystal data

**Table:**

C_21_H_25_NO_2_S	*F*(000) = 760
*M**_r_* = 355.48	*D*_x_ = 1.215 Mg m^−^^3^
Orthorhombic, *P*2_1_2_1_2_1_	Cu *K*α radiation, λ = 1.54178 Å
Hall symbol: P 2ac 2ab	Cell parameters from 9921 reflections
*a* = 6.0621 (1) Å	θ = 3.5–68.2°
*b* = 17.2963 (3) Å	µ = 1.58 mm^−^^1^
*c* = 18.5398 (3) Å	*T* = 100 K
*V* = 1943.93 (6) Å^3^	Block, colourless
*Z* = 4	0.50 × 0.23 × 0.21 mm

### Data collection

**Table:**

Bruker SMART APEX diffractometer	3008 independent reflections
Radiation source: fine-focus sealed tube	2887 reflections with *I* > 2σ(*I*)
Graphite monochromator	*R*_int_ = 0.026
Detector resolution: 8.33 pixels mm^-1^	θ_max_ = 68.3°, θ_min_ = 3.5°
combination of ω and φ–scans	*h* = −7→5
Absorption correction: numerical (*SADABS*; Sheldrick, 2008)	*k* = −20→19
*T*_min_ = 0.720, *T*_max_ = 0.964	*l* = −22→19
18292 measured reflections	

### Refinement

**Table:**

Refinement on *F*^2^	Secondary atom site location: difference Fourier map
Least-squares matrix: full	Hydrogen site location: inferred from neighbouring sites
*R*[*F*^2^ > 2σ(*F*^2^)] = 0.027	H-atom parameters constrained
*wR*(*F*^2^) = 0.071	*w* = 1/[σ^2^(*F*_o_^2^) + (0.0409*P*)^2^ + 0.4225*P*] where *P* = (*F*_o_^2^ + 2*F*_c_^2^)/3
*S* = 1.06	(Δ/σ)_max_ = 0.003
3008 reflections	Δρ_max_ = 0.19 e Å^−^^3^
230 parameters	Δρ_min_ = −0.16 e Å^−^^3^
0 restraints	Absolute structure: Flack (1983), 1165 Friedel pairs
Primary atom site location: structure-invariant direct methods	Flack parameter: 0.039 (15)

### Special details

**Table:**

Geometry. All e.s.d.'s (except the e.s.d. in the dihedral angle between two l.s. planes) are estimated using the full covariance matrix. The cell e.s.d.'s are taken into account individually in the estimation of e.s.d.'s in distances, angles and torsion angles; correlations between e.s.d.'s in cell parameters are only used when they are defined by crystal symmetry. An approximate (isotropic) treatment of cell e.s.d.'s is used for estimating e.s.d.'s involving l.s. planes.
Refinement. Refinement of *F*^2^ against ALL reflections. The weighted *R*-factor *wR* and goodness of fit *S* are based on *F*^2^, conventional *R*-factors *R* are based on *F*, with *F* set to zero for negative *F*^2^. The threshold expression of *F*^2^ > σ(*F*^2^) is used only for calculating *R*-factors(gt) *etc*. and is not relevant to the choice of reflections for refinement. *R*-factors based on *F*^2^ are statistically about twice as large as those based on *F*, and *R*- factors based on ALL data will be even larger.

### Fractional atomic coordinates and isotropic or equivalent isotropic displacement parameters (Å^2^)

**Table:**

	*x*	*y*	*z*	*U*_iso_*/*U*_eq_	
S1	0.98999 (7)	0.85676 (3)	0.12949 (2)	0.02269 (12)	
O1	1.4437 (2)	0.84260 (8)	0.20523 (7)	0.0278 (3)	
O2	1.2940 (2)	0.88301 (7)	0.30979 (6)	0.0219 (3)	
N1	1.0729 (2)	0.87582 (9)	0.21499 (7)	0.0209 (3)	
C1	1.2833 (3)	0.86441 (11)	0.23819 (9)	0.0215 (4)	
C2	0.9378 (3)	0.91505 (11)	0.26982 (8)	0.0201 (4)	
H2A	0.7868	0.8918	0.2706	0.024*	
C3	1.0683 (3)	0.88855 (11)	0.33765 (9)	0.0204 (4)	
C4	0.8825 (3)	0.75865 (11)	0.13766 (10)	0.0262 (4)	
H4A	0.7905	0.7549	0.1823	0.031*	
C5	1.0669 (3)	0.69929 (12)	0.14156 (11)	0.0344 (5)	
H5A	1.0033	0.6473	0.1440	0.052*	
H5B	1.1563	0.7087	0.1847	0.052*	
H5C	1.1600	0.7036	0.0985	0.052*	
C6	0.7353 (4)	0.74711 (14)	0.07179 (11)	0.0376 (5)	
H6A	0.6752	0.6945	0.0722	0.056*	
H6B	0.8223	0.7549	0.0278	0.056*	
H6C	0.6140	0.7845	0.0731	0.056*	
C7	0.9201 (3)	1.00311 (11)	0.25872 (9)	0.0213 (4)	
H7A	0.8672	1.0257	0.3053	0.026*	
C8	1.1389 (3)	1.04238 (12)	0.24058 (10)	0.0283 (4)	
H8A	1.2449	1.0330	0.2795	0.042*	
H8B	1.1153	1.0981	0.2352	0.042*	
H8C	1.1969	1.0212	0.1954	0.042*	
C9	0.7481 (3)	1.02356 (12)	0.20090 (10)	0.0303 (5)	
H9A	0.6098	0.9964	0.2112	0.045*	
H9B	0.8029	1.0079	0.1534	0.045*	
H9C	0.7218	1.0795	0.2012	0.045*	
C10	1.0041 (3)	0.80762 (10)	0.36471 (8)	0.0215 (4)	
C11	1.1590 (3)	0.76845 (12)	0.40647 (10)	0.0275 (5)	
H11A	1.2999	0.7909	0.4145	0.033*	
C12	1.1099 (4)	0.69714 (12)	0.43639 (10)	0.0345 (5)	
H12A	1.2176	0.6709	0.4644	0.041*	
C13	0.9050 (4)	0.66396 (12)	0.42565 (10)	0.0327 (5)	
H13A	0.8709	0.6152	0.4464	0.039*	
C14	0.7502 (3)	0.70267 (12)	0.38436 (10)	0.0300 (5)	
H14A	0.6095	0.6800	0.3765	0.036*	
C15	0.7983 (3)	0.77417 (11)	0.35432 (9)	0.0249 (4)	
H15A	0.6900	0.8004	0.3265	0.030*	
C16	1.0661 (3)	0.94454 (11)	0.40064 (9)	0.0205 (4)	
C17	0.8725 (3)	0.95254 (12)	0.44058 (9)	0.0250 (4)	
H17A	0.7449	0.9241	0.4271	0.030*	
C18	0.8645 (3)	1.00158 (12)	0.49972 (9)	0.0287 (5)	
H18A	0.7311	1.0070	0.5261	0.034*	
C19	1.0499 (3)	1.04269 (12)	0.52041 (9)	0.0299 (5)	
H19A	1.0454	1.0756	0.5614	0.036*	
C20	1.2420 (3)	1.03529 (11)	0.48068 (9)	0.0266 (4)	
H20A	1.3690	1.0639	0.4943	0.032*	
C21	1.2520 (3)	0.98667 (11)	0.42123 (9)	0.0249 (4)	
H21A	1.3852	0.9821	0.3946	0.030*	

### Atomic displacement parameters (Å^2^)

**Table:**

	*U*^11^	*U*^22^	*U*^33^	*U*^12^	*U*^13^	*U*^23^
S1	0.0304 (2)	0.0230 (3)	0.01463 (18)	−0.00277 (19)	−0.00080 (17)	0.00131 (18)
O1	0.0247 (7)	0.0330 (9)	0.0257 (6)	0.0008 (5)	0.0016 (5)	−0.0044 (6)
O2	0.0207 (6)	0.0255 (8)	0.0193 (5)	0.0003 (5)	−0.0009 (5)	−0.0012 (5)
N1	0.0228 (8)	0.0238 (9)	0.0160 (6)	0.0004 (6)	0.0001 (5)	−0.0017 (7)
C1	0.0253 (9)	0.0182 (11)	0.0208 (8)	−0.0043 (8)	0.0007 (7)	−0.0007 (8)
C2	0.0231 (9)	0.0195 (10)	0.0177 (8)	−0.0017 (7)	−0.0001 (6)	−0.0002 (8)
C3	0.0196 (9)	0.0220 (11)	0.0196 (8)	0.0000 (7)	0.0006 (6)	0.0008 (8)
C4	0.0327 (11)	0.0224 (11)	0.0236 (8)	−0.0059 (8)	0.0022 (8)	−0.0020 (9)
C5	0.0399 (12)	0.0245 (12)	0.0389 (11)	0.0001 (9)	−0.0032 (9)	0.0033 (10)
C6	0.0386 (12)	0.0356 (13)	0.0386 (11)	0.0019 (10)	−0.0097 (9)	−0.0143 (10)
C7	0.0257 (9)	0.0209 (11)	0.0173 (7)	0.0008 (7)	−0.0006 (7)	0.0011 (8)
C8	0.0340 (11)	0.0229 (11)	0.0280 (9)	−0.0017 (8)	0.0006 (8)	0.0064 (9)
C9	0.0315 (11)	0.0273 (13)	0.0320 (9)	0.0032 (8)	−0.0049 (8)	0.0033 (9)
C10	0.0318 (9)	0.0180 (10)	0.0148 (7)	0.0020 (8)	0.0030 (8)	−0.0020 (7)
C11	0.0350 (11)	0.0249 (12)	0.0227 (8)	0.0008 (8)	−0.0038 (8)	0.0019 (9)
C12	0.0510 (13)	0.0273 (13)	0.0252 (9)	0.0087 (10)	−0.0037 (9)	0.0036 (10)
C13	0.0575 (13)	0.0169 (12)	0.0236 (9)	0.0020 (9)	0.0091 (9)	0.0017 (9)
C14	0.0382 (11)	0.0240 (12)	0.0277 (9)	−0.0029 (8)	0.0068 (8)	−0.0015 (9)
C15	0.0291 (10)	0.0218 (11)	0.0237 (8)	0.0009 (8)	0.0024 (7)	0.0006 (9)
C16	0.0269 (10)	0.0179 (10)	0.0167 (7)	0.0017 (7)	−0.0033 (6)	0.0044 (8)
C17	0.0284 (10)	0.0232 (11)	0.0234 (9)	0.0005 (8)	−0.0008 (7)	0.0035 (9)
C18	0.0401 (11)	0.0256 (12)	0.0204 (8)	0.0071 (9)	0.0045 (8)	0.0010 (9)
C19	0.0483 (13)	0.0224 (11)	0.0190 (8)	0.0074 (9)	−0.0063 (8)	−0.0014 (9)
C20	0.0367 (11)	0.0187 (11)	0.0244 (9)	−0.0006 (8)	−0.0117 (8)	−0.0003 (8)
C21	0.0298 (10)	0.0217 (11)	0.0231 (8)	0.0016 (8)	−0.0035 (7)	0.0023 (9)

### Geometric parameters (Å, º)

**Table:**

S1—N1	1.6952 (14)	C20—C21	1.388 (3)
S1—C4	1.8240 (19)	C2—H2A	1.0000
O1—C1	1.209 (2)	C4—H4A	1.0000
O2—C1	1.367 (2)	C5—H5A	0.9800
O2—C3	1.466 (2)	C5—H5B	0.9800
N1—C1	1.361 (2)	C5—H5C	0.9800
N1—C2	1.471 (2)	C6—H6A	0.9800
C2—C7	1.541 (3)	C6—H6B	0.9800
C2—C3	1.555 (2)	C6—H6C	0.9800
C3—C16	1.517 (3)	C7—H7A	1.0000
C3—C10	1.537 (3)	C8—H8A	0.9800
C4—C5	1.519 (3)	C8—H8B	0.9800
C4—C6	1.525 (3)	C8—H8C	0.9800
C7—C8	1.528 (3)	C9—H9A	0.9800
C7—C9	1.537 (2)	C9—H9B	0.9800
C10—C15	1.388 (3)	C9—H9C	0.9800
C10—C11	1.393 (3)	C11—H11A	0.9500
C11—C12	1.385 (3)	C12—H12A	0.9500
C12—C13	1.383 (3)	C13—H13A	0.9500
C13—C14	1.384 (3)	C14—H14A	0.9500
C14—C15	1.387 (3)	C15—H15A	0.9500
C16—C17	1.395 (2)	C17—H17A	0.9500
C16—C21	1.395 (3)	C18—H18A	0.9500
C17—C18	1.387 (3)	C19—H19A	0.9500
C18—C19	1.385 (3)	C20—H20A	0.9500
C19—C20	1.384 (3)	C21—H21A	0.9500
			
N1—S1—C4	102.07 (8)	C4—C5—H5B	109.5
C1—O2—C3	108.25 (12)	H5A—C5—H5B	109.5
C1—N1—C2	111.72 (13)	C4—C5—H5C	109.5
C1—N1—S1	123.04 (12)	H5A—C5—H5C	109.5
C2—N1—S1	124.82 (11)	H5B—C5—H5C	109.5
O1—C1—N1	129.76 (16)	C4—C6—H6A	109.5
O1—C1—O2	121.74 (15)	C4—C6—H6B	109.5
N1—C1—O2	108.50 (14)	H6A—C6—H6B	109.5
N1—C2—C7	113.74 (14)	C4—C6—H6C	109.5
N1—C2—C3	98.05 (13)	H6A—C6—H6C	109.5
C7—C2—C3	115.78 (14)	H6B—C6—H6C	109.5
O2—C3—C16	108.72 (14)	C8—C7—H7A	107.1
O2—C3—C10	106.96 (14)	C9—C7—H7A	107.1
C16—C3—C10	109.14 (14)	C2—C7—H7A	107.1
O2—C3—C2	102.07 (12)	C7—C8—H8A	109.5
C16—C3—C2	115.48 (15)	C7—C8—H8B	109.5
C10—C3—C2	113.81 (15)	H8A—C8—H8B	109.5
C5—C4—C6	112.33 (16)	C7—C8—H8C	109.5
C5—C4—S1	111.71 (13)	H8A—C8—H8C	109.5
C6—C4—S1	105.32 (14)	H8B—C8—H8C	109.5
C8—C7—C9	109.46 (15)	C7—C9—H9A	109.5
C8—C7—C2	114.11 (15)	C7—C9—H9B	109.5
C9—C7—C2	111.60 (15)	H9A—C9—H9B	109.5
C15—C10—C11	118.71 (17)	C7—C9—H9C	109.5
C15—C10—C3	124.17 (16)	H9A—C9—H9C	109.5
C11—C10—C3	116.97 (17)	H9B—C9—H9C	109.5
C12—C11—C10	120.71 (19)	C12—C11—H11A	119.6
C13—C12—C11	120.3 (2)	C10—C11—H11A	119.6
C12—C13—C14	119.21 (19)	C13—C12—H12A	119.8
C13—C14—C15	120.7 (2)	C11—C12—H12A	119.8
C14—C15—C10	120.31 (18)	C12—C13—H13A	120.4
C17—C16—C21	118.85 (17)	C14—C13—H13A	120.4
C17—C16—C3	118.63 (16)	C13—C14—H14A	119.6
C21—C16—C3	122.49 (15)	C15—C14—H14A	119.6
C18—C17—C16	120.67 (18)	C14—C15—H15A	119.8
C19—C18—C17	120.29 (18)	C10—C15—H15A	119.8
C18—C19—C20	119.23 (17)	C18—C17—H17A	119.7
C19—C20—C21	121.05 (19)	C16—C17—H17A	119.7
C20—C21—C16	119.90 (18)	C19—C18—H18A	119.9
N1—C2—H2A	109.6	C17—C18—H18A	119.9
C7—C2—H2A	109.6	C18—C19—H19A	120.4
C3—C2—H2A	109.6	C20—C19—H19A	120.4
C5—C4—H4A	109.1	C19—C20—H20A	119.5
C6—C4—H4A	109.1	C21—C20—H20A	119.5
S1—C4—H4A	109.1	C20—C21—H21A	120.0
C4—C5—H5A	109.5	C16—C21—H21A	120.0
			
C4—S1—N1—C1	94.18 (16)	C16—C3—C10—C15	103.39 (18)
C4—S1—N1—C2	−93.83 (15)	C2—C3—C10—C15	−27.2 (2)
C2—N1—C1—O1	−170.44 (19)	O2—C3—C10—C11	45.47 (19)
S1—N1—C1—O1	2.5 (3)	C16—C3—C10—C11	−71.98 (19)
C2—N1—C1—O2	8.9 (2)	C2—C3—C10—C11	157.40 (15)
S1—N1—C1—O2	−178.19 (11)	C15—C10—C11—C12	0.7 (3)
C3—O2—C1—O1	−166.32 (17)	C3—C10—C11—C12	176.36 (16)
C3—O2—C1—N1	14.3 (2)	C10—C11—C12—C13	−0.5 (3)
C1—N1—C2—C7	96.89 (17)	C11—C12—C13—C14	0.3 (3)
S1—N1—C2—C7	−75.89 (17)	C12—C13—C14—C15	−0.4 (3)
C1—N1—C2—C3	−25.94 (18)	C13—C14—C15—C10	0.7 (3)
S1—N1—C2—C3	161.28 (13)	C11—C10—C15—C14	−0.8 (3)
C1—O2—C3—C16	−152.32 (15)	C3—C10—C15—C14	−176.10 (16)
C1—O2—C3—C10	89.95 (16)	O2—C3—C16—C17	−173.21 (15)
C1—O2—C3—C2	−29.84 (18)	C10—C3—C16—C17	−56.9 (2)
N1—C2—C3—O2	31.85 (15)	C2—C3—C16—C17	72.8 (2)
C7—C2—C3—O2	−89.47 (16)	O2—C3—C16—C21	5.0 (2)
N1—C2—C3—C16	149.59 (15)	C10—C3—C16—C21	121.36 (18)
C7—C2—C3—C16	28.3 (2)	C2—C3—C16—C21	−108.93 (19)
N1—C2—C3—C10	−83.01 (16)	C21—C16—C17—C18	0.1 (3)
C7—C2—C3—C10	155.67 (14)	C3—C16—C17—C18	178.38 (17)
N1—S1—C4—C5	−76.73 (14)	C16—C17—C18—C19	−0.7 (3)
N1—S1—C4—C6	161.06 (13)	C17—C18—C19—C20	1.1 (3)
N1—C2—C7—C8	−44.64 (18)	C18—C19—C20—C21	−0.9 (3)
C3—C2—C7—C8	67.84 (19)	C19—C20—C21—C16	0.2 (3)
N1—C2—C7—C9	80.09 (18)	C17—C16—C21—C20	0.2 (3)
C3—C2—C7—C9	−167.43 (14)	C3—C16—C21—C20	−178.05 (17)
O2—C3—C10—C15	−139.16 (16)		
